# TV005 dengue vaccine protects against dengue serotypes 2 and 3 in two controlled human infection studies

**DOI:** 10.1172/JCI173328

**Published:** 2024-02-01

**Authors:** Kristen K. Pierce, Anna P. Durbin, Mary-Claire R. Walsh, Marya Carmolli, Beulah P. Sabundayo, Dorothy M. Dickson, Sean A. Diehl, Stephen S. Whitehead, Beth D. Kirkpatrick

**Affiliations:** 1 Department of Medicine and; 2 Department of Microbiology and Molecular Genetics, The University of Vermont Larner College of Medicine, Vaccine Testing Center, Burlington, Vermont, USA.; 3 The Johns Hopkins Bloomberg School of Public Health, Baltimore, Maryland, USA.; 4 National Institute of Allergy and Infectious Diseases (NIAID), Laboratory of Viral Diseases, Bethesda, Maryland, USA.

**Keywords:** Infectious disease, AIDS vaccine

## Abstract

**BACKGROUND:**

Disease due to dengue viruses is a growing global health threat, causing 100–400 million cases annually. An ideal dengue vaccine should demonstrate durable protection against all 4 serotypes in phase III efficacy trials, however the lack of circulating serotypes may lead to incomplete efficacy data. Controlled human infection models help downselect vaccine candidates and supply critical data to supplement efficacy trials. We evaluated the efficacy of a leading live-attenuated tetravalent dengue vaccine candidate, TV005, against infection with a newly established dengue serotype 3 or an established serotype 2 challenge virus.

**METHODS:**

Two randomized, controlled clinical trials were performed. In study 1, a total of 42 participants received TV005 or placebo (*n* = 21 each), and 6 months later, all were challenged with dengue 2 virus (rDEN2Δ30) at a dose of 10^3^ PFU. In study 2, a total of 23 participants received TV005 and 20 received placebo, and 6 months later, all were challenged with 10^4^ PFU dengue 3 virus (rDEN3Δ30). The study participants were closely monitored for safety, viremia, and immunologic responses. Infection, measured by post-challenge viremia, and the occurrence of rash and neutropenia were the primary endpoints. Secondary endpoints included safety, immunologic, and virologic profiles following vaccination with TV005 and subsequent challenge with the rDEN2Δ30 or rDEN3Δ30 strain.

**RESULTS:**

TV005 was well tolerated and protected all vaccinated volunteers from viremia with DENV2 or DENV3 (none infected in either group). Placebo recipients had post-challenge viremia (100% in study 1, 85% in study 2), and all experienced rash following challenge with either serotype.

**CONCLUSIONS:**

TV005 is a leading tetravalent dengue vaccine candidate that fully protected against infection with DENV2 and DENV3 in an established controlled human infection model.

**TRIAL REGISTRATION:**

ClinicalTrials.gov NCT02317900 and NCT02873260.

**FUNDING:**

Intramural Research Program, NIH (contract HHSN272200900010C).

## Abstract 2

<p>Two randomized, placebo-controlled vaccine-human infection model trials demonstrate complete protective efficacy of the TV005 dengue vaccine against Dengue serotype 2 and 3 infection.</p>

## Introduction

Dengue viruses are estimated to cause 100–400 million symptomatic illnesses each year, with a significant and expanding burden of severe disease and mortality in both endemic populations and travelers (1–4). The geographic distribution of dengue is also expanding (5). All 4 serotypes of dengue viruses (DENV1–4) cause symptomatic disease and can circulate independently or concurrently. Severe disease manifestations include hemorrhagic fever, plasma leakage leading to shock, and death. Disease management is solely supportive; to date, specific therapeutics are not available for use (6).

The development of a vaccine that is safe and effective against all 4 serotypes has been a global priority (7). Importantly, individuals experiencing a second dengue infection with a new serotype appear to be at higher risk for more severe disease. Partial serotype-specific immunity conferred by the first infection may facilitate increased virus entry and enhance the replication of a subsequent serotype. Thus, a successful tetravalent dengue vaccine should concurrently protect against all 4 serotypes.

Dengue vaccines in advanced development are all live-attenuated tetravalent vaccines (LATVs), since the immunogenicity of an LATV is superior to that of other vaccine designs (e.g., inactivated, subunit, DNA) (8). LATVs induce both robust humoral and cellular immunity, present viral epitopes in their native conformation, and are generally less expensive to produce (8). Three LATVs are in late-stage development: the NIH’s dengue vaccines, TV003 and TV005 vaccines (9), and Takeda’s TAK-003 (10). Sanofi Pasteur’s tetravalent vaccine Dengvaxia was first licensed in 2015 in Mexico. Because of observations of increased disease-related hospitalizations in DENV-naive vaccine recipients, it is now recommended by the FDA, the European Medicines Agency, and the WHO for use only in previously dengue-exposed individuals (11). Similar concerns have been raised with the Takeda TAK-003 vaccine (7, 12, 13).

Relevant to this work, the NIH dengue vaccines are attenuated by the deletion of nucleotides from the 3′-UTR of the viral genome (8). These vaccines contain structural and nonstructural proteins derived solely from DENV, with full-length genomes of DENV1, -3, and -4 serotypes and a chimeric genome for the DENV2 serotype. The dengue strains used in the TV005 vaccine are rDEN1Δ30, rDEN2/4Δ30 (New Guinea C [NGC] strain), rDEN3Δ30/31, and rDEN4Δ30. TV003 includes 10^3^ PFU of each serotype. TV005 is identical, but has an increased dosage (10^4^ PFU) of the DENV2 component and was designed after early phase I trials suggested lower immunogenicity to DENV2 (14). Although subsequent clinical trials have eliminated this early concern (15), both TV003 and TV005 have progressed in development toward licensure internationally for further development by multiple pharmaceutical companies (9). The immunogenicity of both vaccines benefits from a transient low-level viremia, as expected for a live vaccine (14). Both formulations elicit cellular immune responses to the nonstructural components and tetravalent neutralizing homotypic antibody responses in the majority of individuals (16–18).

Advanced development of LATVs prompted conversations about what safety and efficacy data should precede and/or supplement large-scale testing of a tetravalent vaccine in dengue-endemic settings (13). To this end, our team used controlled human infection models (CHIMs), which, studied alongside vaccines, are important tools that provide early measures of vaccine efficacy of specific serotypes and complement phase III trials. Notably, if efficacy is not demonstrated using a CHIM, a LATV may be modified or eliminated before reaching large efficacy trials (19). The challenge virus strains themselves (DENV2 [Tonga/74], and DENV3 [Sleman/78]) are derived from dengue outbreaks in Tonga and Indonesia, respectively, and are associated with relatively mild clinical disease, low viremia levels, and few hemorrhagic manifestations and thus can be considered to be naturally attenuated DENV strains (20, 21). The clinical products used in the challenge model are recombinant versions designed from very low-passage isolates that were also engineered to contain the Δ30 mutation with an original goal of using them as vaccine candidates. Studies in nonhuman primates indicated that the rDEN2Δ30 (Tonga/74) and rDEN3Δ30 (Sleman/78) strains were not significantly attenuated compared with their WT parent viruses (22, 23). Although the Δ30 mutation was attenuating for other serotypes/strains of DENV such as DENV1 and DENV4, the Δ30 mutation did not confer an additional attenuation phenotype to DENV2 Tonga/74 or DENV3 Sleman/78, and the Δ30 derivatives were demonstrated in phase I testing to be too reactogenic as vaccine candidates (9).

With the ultimate goal of developing an affordable vaccine for the most at-risk populations, particularly dengue-naive children and adults, NIH tetravalent vaccines have been developed over 2 decades of methodical and iterative studies, beginning with testing of each monovalent vaccine, followed by evaluation of tetravalent vaccine admixtures and, now, human experimental infections (CHIMs) and phase III trials. This process has determined homotypic immunogenicity to all serotypes, inoculum size, use of a single dose, and vaccine safety in individuals up to 70 years of age and has included participants in the United States, Thailand, Brazil, and Bangladesh. In addition, our initial evaluation of TV003 in a CHIM demonstrated complete protection against infection with a DENV2 challenge virus 6 months after vaccination (15).

Here, using 2 randomized, controlled clinical trials, our objectives were to confirm the efficacy of the second NIH tetravalent vaccine (TV005) against DENV2 (rDEN2Δ30, Tonga strain) or DENV3 (rDEN3Δ30/Sleman 78) infection. This work was the first use of our DENV3 attenuated challenge virus to test the efficacy of any LATV. Our secondary objectives included safety, immunologic, and virologic profiles of the participants receiving TV005 and the subsequent rDEN2Δ30 or rDEN3Δ30 challenge strains.

## Results

**Figure 1:**
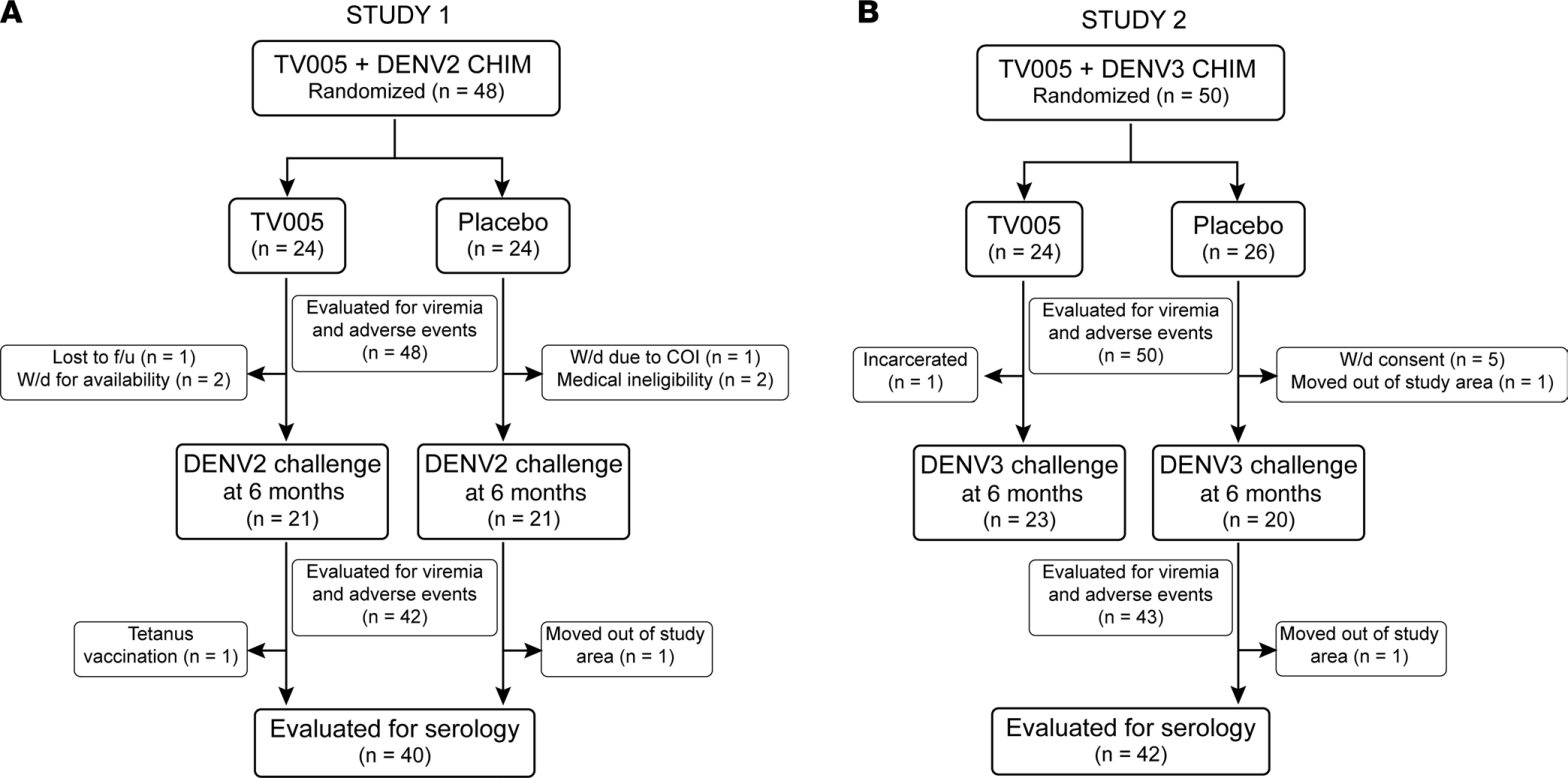
CONSORT diagrams of enrollment, retention, and interventions. (**A**) Study 1 (CIR299) is a placebo-controlled trial of the efficacy of TV005 against rDEN2Δ30 challenge. (**B**) Study 2 (CIR309) is a placebo-controlled trial of the efficacy of TV005 against rDEN3Δ30 challenge. COI, conflict of interest; f/u, follow-up; W/d, withdrew.

Following screening, 319 participants were evaluated for participation, and 98 were randomized to receive TV005 vaccine or placebo (*n* = 48 in study 1 and *n* = 50 in study 2) and were monitored as described in the Methods. Virus challenge of all participants occurred 6 months after vaccination, in both studies. Forty-two participants in study 1 (21 each received either TV005 or placebo) were given the rDEN2Δ30 challenge virus. In study 2, a total of 43 participants (23 received TV005 and 20 received placebo) were given the rDEN3Δ30 challenge virus. As noted in Figure 1, we removed 13 participants from the study before virus challenge for medical ineligibility, incarceration, conflict of interest, or withdrawal of consent; 2 participants in study 2 were replaced, as they withdrew prior to study day 28 (kept for safety evaluation).

Demographic data for participants in both studies are shown in Table 1; significant differences were not noted between the groups when age, sex, and race were evaluated.

### Measures of efficacy.

As shown in Table 2, the TV005 vaccine protected all vaccinated individuals from both viremia (primary efficacy endpoint) and rash following DENV2 or DENV3 challenge 6 months after vaccination with TV005. In contrast, in participants who did not receive the TV005 vaccine and who subsequently received the DENV2 challenge virus, all individuals had DENV2 viremia (*n* = 21, 100%). For placebo recipients who subsequently received the DENV3 challenge strain, 17 (85%) had DENV3 viremia.

Viremia titers after vaccination and challenge are shown in Table 3. Following challenge, DENV2 viremia by direct culturing had a mean peak titer of 2.23 log_10_ PFU/mL and a maximum peak titer of 3.2 log_10_ PFU/mL, with a mean duration of 4.6 (range, 2–6) days. DENV3 challenge viremia was lower (mean peak titer of 1.07 log_10_ PFU/mL, maximum 2.2 log_10_ PFU/mL) and a shorter mean duration of 2 (range, 1–5) days.

Following DENV2 or DENV3 virus challenge, all (100%) placebo recipients in both studies had a rash. Neutropenia (defined as an absolute neutrophil count [ANC] of <1,000 cells/mm^3^) was not a statistically significant marker of vaccine efficacy.

### Safety and reactogenicity to TV005 vaccination.

Adverse events (AEs) were minimal and are shown in Table 4. As anticipated based on past studies, significant associations with vaccination were mild rash in 30 (62.5%) vaccinees, injection site erythema in 6 (12.5%) vaccinees, and a mild, short-lived neutropenia in 6 (12.5%) vaccinees.

### Safety and reactogenicity to viral challenges.

**Figure 2:**
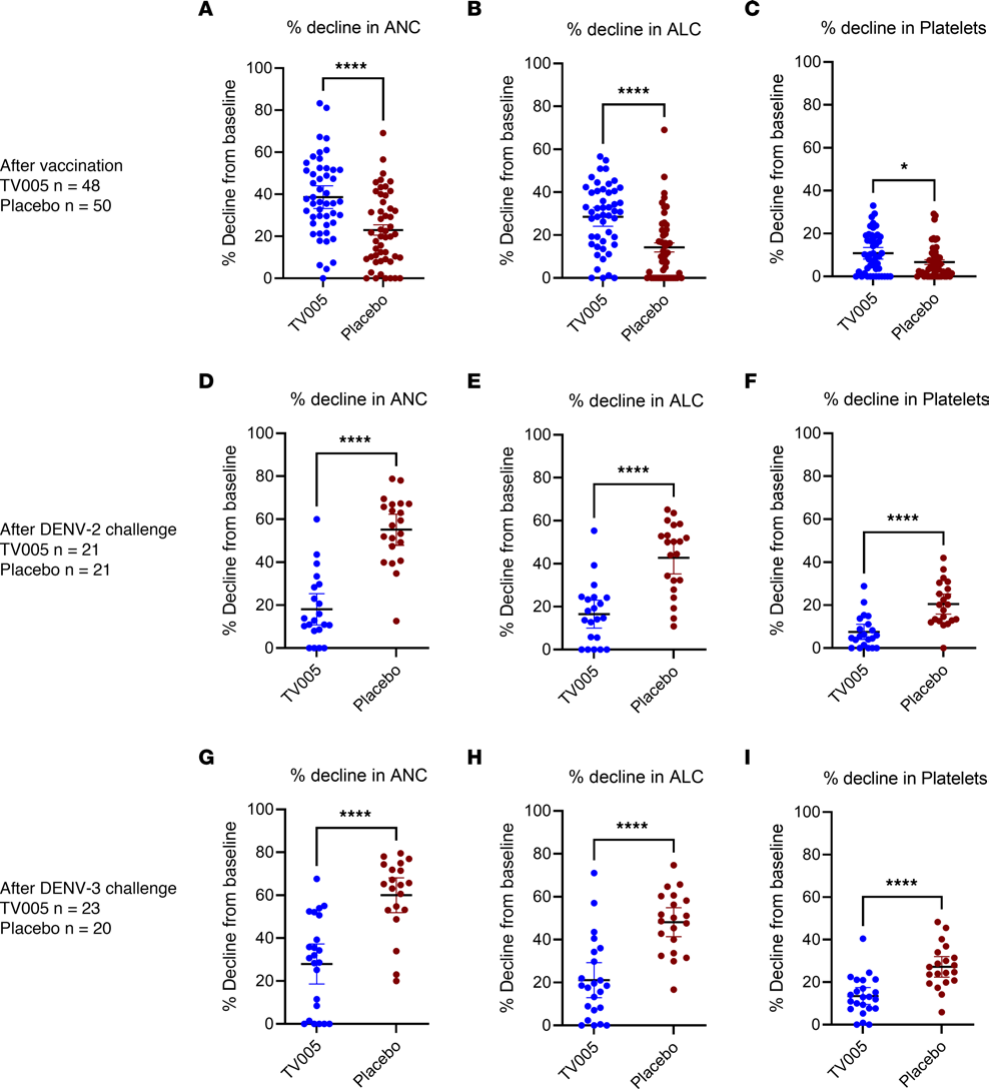
Decline in ALC, ANC, and platelets following administration of TV005 vaccine and DENV2 or DENV3 challenge 6 months later. Percentage of decline of the ANC, ALC, and platelet count following vaccination with TV005 compared with placebo. All TV005-vaccinated participants (*n* = 48) and placebo recipients (*n* = 50) from both CIR299 and CIR309 were included in these analyses. The nadir of neutrophil, lymphocyte, and platelet counts, measured through study day 16 after vaccination (or challenge), was compared with the value at study day 0 before vaccination (or before challenge). The percentage of decline was calculated as the absolute decline/baseline value × 100. Individual data along with means and 95% CIs are shown. An unpaired, 2-tailed Student’s *t* test was used to compare TV005 versus placebo. (**A**–**C**) Percentage of decline of the ANC (**A**), ALC (**B**), and absolute platelet count (**C**) after vaccination compared with the values prior to vaccination (day 0) for volunteers who received TV005 vaccination or placebo. The percentage of decline was greater among TV005 participants than among placebo recipients (neutrophils and lymphocytes *P* < 0.0001, platelets *P* = 0.018). (**D**–**F**) Percentage of decline of the ANC (**D**), ALC (**E**), and absolute platelet count (**F**) compared with the day of virus challenge in participants who received TV005 or placebo and who were all subsequently (6 months later) challenged with DENV2 virus. Volunteers who received placebo (not the TV005 vaccine) had significantly greater declines in neutrophils, lymphocytes, and platelets following DENV2 challenge compared with volunteers who received TV005 (*P* < 0.0001). (**G**–**I**) Percentage of decline of the ANC (**G**), ALC (**H**), and absolute platelet count (**I**) compared with the day of viral challenge in participants who received TV005 or placebo and who, 6 months later, all received DENV3 virus challenge. Volunteers who received placebo (not TV005 vaccine) had significantly greater declines in neutrophils, lymphocytes, and platelets following DENV3 challenge compared with volunteers who received TV005 (*P* < 0.0001). *P < 0.05 and ****P < 0.0001, by unpaired, 2-tailed Student’s *t* test (**A**–**I**).

Following receipt of the DENV2 challenge strain (Table 5 and Figure 2), AEs were noted predominately among the placebo recipients, including myalgias and retro-orbital pain and cytopenia. An ANC of less than 1,000 cells/mm^3^ did not differ between groups, but significant differences were noted regarding the percentage of ANC decline, as well as for lymphocytes and platelets. Likewise, as seen in Table 6 and Figure 2, AEs following the DENV3 challenge predominated in the placebo group.

We compared the AEs following DENV2 and DENV3 challenge, including participant data from 2 previous rDEN2Δ30 CHIM studies (15, 19) and 1 previous rDEN3Δ30 CHIM study (24). All virus challenges were performed 6 months after vaccination. As shown in Table 7, and despite the lower viremia (as above), the DENV3 CHIM was associated with significantly more local AEs (erythema, induration, tenderness) at the injection site, as well as systemic symptoms of fatigue and headache, compared with the DENV2 CHIM. Fever was not noted in either CHIM, with the exception of a single DENV3 challenge volunteer, and no significant differences were found in the occurrence of cytopenias or in liver function tests. The severity and duration of rash in the 2 models are detailed in Table 8.

### Serologic responses.

**Figure 3:**
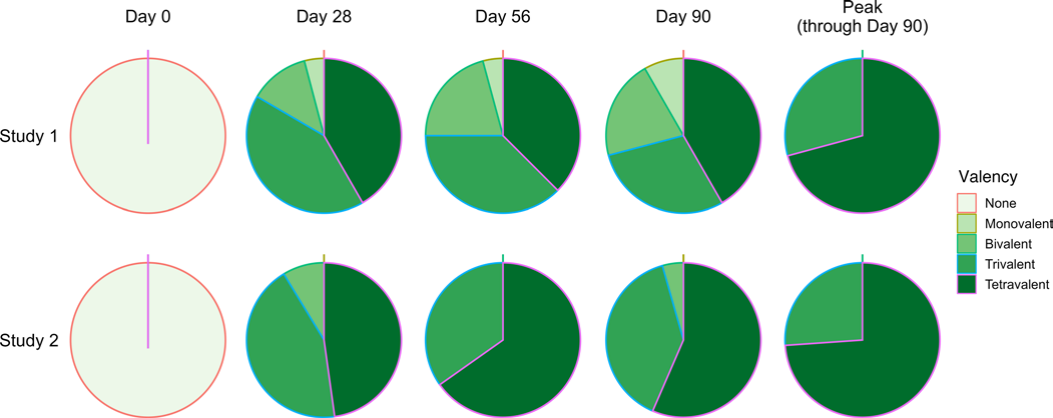
Seroconversion to multiple serotypes following vaccination of dengue-naive adult volunteers with TV005. Following TV005 vaccination of adult dengue-naive volunteers (day) and at subsequent time points after vaccination, volunteers demonstrated seroconversion to 1 (monovalent) to 4 (tetravalent) serotypes. The majority of participants in study 1 (CIR299) and study 2 (CIR309) demonstrated seroconversion to all 4 (trivalent) serotypes.

Following TV005 vaccination, with data from both studies combined, we found neutralizing antibody responses consistent with seroconversion and seropositivity to all serotypes in the majority of vaccine recipients — from 79% (DENV1) to 100% (DENV2) (Table 9). We found that 100% of the vaccinees had serologic responses to 3 serotypes (trivalent response), and 72% had a tetravalent response (Figure 3)

In study 1 (Table 10), 18 (90%) of placebo recipients who received the DENV2 challenge became seropositive for the rDEN2Δ30 Tonga/74 challenge strain by study day 90; 18 (90%) also became seropositive for the vaccine homotypic DENV2 NGC strain. By day 180 after challenge, all 20 participants were seropositive for both DENV2 strains. Interestingly, geometric mean titers (GMTs) were higher with the NGC strain (GMT of 284) compared with the Tonga strain (GMT of 61). In the placebo recipient group, heterotypic cross-reactive antibody responses to the other serotypes (serotypes 1, 3, and 4) were modest. For placebo recipients subsequently receiving the DENV3 challenge, 19 volunteers (100%) became seropositive, with a GMT of 417 (GMT range, 15–1,932). Interestingly, in this group, we observed more heterotypic cross-reactive antibodies, with concurrent seropositivity in 47% of participants to DENV1 (GMT of 38), 79% to DENV2 NGC (GMT of 61), and 21% to DENV4 (GMT of 27).

**Figure 4:**
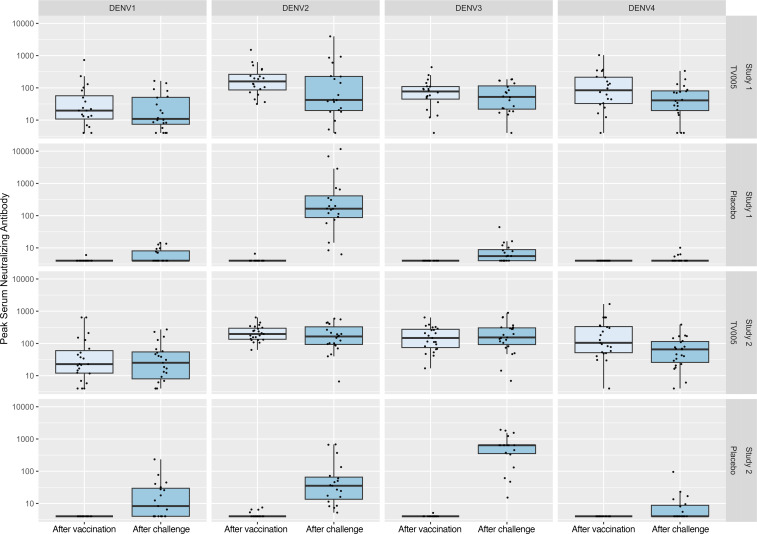
Serum neutralizing antibody responses through day 90 in TV005 vaccine or placebo recipients, following TV005 vaccination and subsequent DEVN2 or DEVN3 virus challenge. Individual data are shown along with box and whisker plots; the box is the median with 25th and 75th percentiles; whiskers extend to the minimum/maximum values, no further than 1.5 times the IQR from the lower/upper quartile. For DENV2 responses, all data shown after vaccination and challenge reflect responses to the rDEN2/4Δ30 (NGC) strain. Responses to both the rDEN2Δ30 (NGC) and rDEN2Δ30 strain (Tonga) are shown in Table 10.

For individuals who received the TV005 vaccine, seropositivity and GMT did not change markedly following viral challenge with DENV2 or -3, and boosting (of seropositivity or GMT) was not observed (Table 10 and Figure 4).

## Discussion

Leveraging CHIMs that use DENV2 and DENV3 challenge viral strains, we demonstrate that the NIH live-attenuated tetravalent dengue vaccine TV005 provided complete (100%) protective efficacy against infection with DENV2 or DENV3 six months after vaccination of dengue-naive individuals. The use of CHIM allowed us to assess vaccine efficacy from known serotypes using controlled inoculum, a known time of infection, and close clinical observation.

This work represents the first CHIM-based demonstration of efficacy of the tetravalent TV005 vaccine to prevent infection with DENV2 and DENV3. Furthermore, this is the first report of vaccine efficacy testing using the rDEN3Δ30 CHIM and the largest study thus far in which a DENV3 challenge strain was used.

Both rDEN2Δ30 and rDEN3Δ30 challenge models were tested for safety in advance of this work (15, 24). Both CHIMs are designed as “infection models” with an endpoint of viremia and mild symptoms of dengue infection, including mild-to-moderate rash, transient neutropenia, and mild retro-orbital pain, all prevented by TV005 vaccination. In contrast, CHIM strains under development by other teams are designed as “disease models” and cause signs and symptoms consistent with classic dengue fever including fever and high viremia (19, 25, 26). We believe the protective effect of TV005 against infection and concomitant symptoms of rDEN2Δ30 and rDEN3Δ30 “infection models” are indicative of protection against WT dengue and safe for human volunteers. The DENV2 component of the work herein builds on and confirms our previous evaluation of the NIH TV003 vaccine (15), which contains a 10-fold lower dosage of the DENV2 vaccine component (10^3^ PFU) compared with TV005 (27). Both TV003 and TV005 protected all (100%) individuals from infection with rDEN2Δ30. During the development of TV005, neutralization against parent virus strain targets provided an unbiased means of evaluating immunogenicity, since it is possible to find target strains that yield either higher or lower titers compared with the actual parental strain (28). Nonetheless, we note that the DENV2 vaccine component strain (NGC/1944 [an Asian II genotype]) elicited neutralizing antibodies against the American genotype Tonga/1974 challenge virus as well as other more recent strains (29).

We believe this work is an important milestone in the late stages of human testing of the TV003 and TV005 vaccines and adds to the data set a demonstration of safety and broad serotype immunogenicity of TV005, including in endemic settings (9, 14, 30). TV003 is being tested in ongoing phase III vaccine efficacy evaluations in Brazil. Two-year data suggest an overall vaccine efficacy rate of 80% against the 2 circulating strains (DENV1, 90% and DENV2, 70%) (31). Notably, efficacy is not 100%, as was the case in CHIMs 6 months after vaccination (15). Multiple factors found in a real-world field trial can explain the lack of precision between the models but do not diminish the power of the CHIM as an initial evaluation of vaccine efficacy. These factors include local burden/intensity of transmission, infection by a mosquito rather than a needle, time interval between vaccination and DENV exposure, repeated exposures, and circulating strains. Our continued understanding of these variables will improve dengue CHIMs. Critically, the absence of circulation of DENV3 and DENV4 serotypes in this phase III trial (and thus the lack of serotype efficacy data) underscores the vital importance of CHIMs to test vaccines against these missing serotypes, such as DENV3. Our DENV4 challenge virus is currently under development.

The opportunities for DENV CHIMs as an integrated component of global dengue vaccine development are still being realized. Additional dengue serotypes and strains from different viral backgrounds have been described (25, 26, 32, 33); each model has distinct features, including various degrees of viral titer (viremia) and clinical signs and symptoms. Of particular interest are opportunities for vaccine CHIMs in dengue-endemic settings in which the documentation of efficacy in populations with varying degrees of baseline immunity would increase confidence before the large-scale introduction of dengue vaccines or phase III efficacy trials in new populations. TV005 DENV challenge models (DENV2 and DENV3) are ongoing in Dhaka, Bangladesh. As noted above, CHIMs may supply critical data to assess vaccine efficacy for serotypes that are not continuously circulating in a specific population. They may also be used to clinically interrogate specific viral infection and replication processes, especially those that occur very early after infection and are clinically unapparent in natural settings.

This work has notable limitations and observations that are not yet explained. We observed a disconnect between viremia and reactogenicity in our DENV3 CHIM. The rDEN3Δ30 challenge was provided here at a 10^4^ PFU dose and had lower viremia than that observed in the DENV2 CHIM (10^3^ PFU). Conversely, despite low viremia, this DENV3 model had more reactogenicity (including rash) than was seen in volunteers receiving the rDEN2Δ30 challenge strain. In addition, and possibly explained by innate immune responses, the higher dose (10^4^ PFU) of rDEN3Δ30 infected only 85% of placebo recipients versus 100% of those receiving the lower (10^3^ PFU) dose in a previous trial (24, 34). It is hoped that planned work on cellular and innate immune contributions in these cohorts will clarify these observations.

In addition, human infection models cannot fully replace field testing in phase III efficacy and phase IV post-licensure evaluations of a new tetravalent dengue vaccine. These models cannot represent all possible dengue strains (and their intra-serotype breadth) or predict the duration of protection, nor can they expand the safety database through the study of a large population. Our models were studied 6 months after vaccination, which may not be comparable to efficacy trials that assess protection over multiple years. CHIMs cannot ethically be used in children. However, as models, they offer significant advances in our understanding of the safety, immunogenicity, and efficacy of new dengue vaccines. Given the infectivity of all 4 serotype components, TV005 provides homotypic neutralizing antibodies to all 4 serotypes, as well as cellular immune responses to nonstructural proteins and has now been demonstrated to provide complete protection against both DENV3 and DENV2 challenge.

## Methods

Two randomized, placebo-controlled, vaccine CHIM clinical trials were performed using the NIH’s live-attenuated tetravalent dengue vaccine admixture TV005, under protocols CIR 299 (study 1, DENV2) and CIR 309 (study 2, DENV3). Trials were conducted in Burlington, Vermont, and Baltimore, Maryland, using good clinical practice standards.

The TV005 vaccine contains 10^3^ PFU each of DENV1, -3, and -4 and 10^4^ PFU of DENV2. The attenuated DENV strains used in the TV005 vaccine were rDEN1Δ30 (WestPac/74), rDEN2/4Δ30 (NGC), rDEN3Δ30/31 (Sleman/78), and rDEN4Δ30 (Dominica/81). Attenuation of these vaccine strains is medicated by the Δ30 mutation (and an additional Δ31 for DEN3Δ30/31 and by chimerization in the case of rDEN2/4Δ30). The rDEN2Δ30 and rDEN3Δ30 strains used as challenge virus were rDEN2Δ30 (Tonga/74) and rDEN3Δ30 (Sleman/78), respectively, both underattenuated for use as vaccine components (19, 24).

The primary objective of each trial was vaccine efficacy in individuals who had received either TV005 vaccine or placebo and who subsequently received challenge with DENV2 or DENV3 at 6 months. The primary efficacy endpoint was the presence or absence of challenge-virus viremia, measured at any point following administration of the DENV2 or DENV3 virus challenge strain.

A second measure of efficacy was the presence of rash and/or neutropenia following the DENV2 or DENV3 viral challenges. Additional study objectives included detailed assessments of serologic responses (peak, geometric mean titers, change following virus challenge, and comparison between studies) and viremia (i.e., frequency, magnitude [maximum titer], and duration).

Inclusion and exclusion criteria included generally healthy dengue-naive men and nonpregnant women, aged 18–50 years. Participants were enrolled following comprehensive screening. All were seronegative for HIV, hepatitis B and C, had normal hematology and blood chemistry results, and were otherwise generally healthy as determined by history and physical examination. Women of reproductive age were required to use approved contraceptive methods for the duration of the study.

For both clinical trials, individuals were randomized 1:1 in 8 blocks of 6. Each block included 3 vaccine recipients and 3 placebo recipients. A master log of treatment assignments was maintained separately from the study records, and a sealed copy of the treatment assignments was given to the Data Safety Monitoring Board (DSMB) Executive Secretary. Investigators and all clinical staff remained blinded to the treatments until all participants in a treatment block reached study day 270.

Six months after receipt of either the vaccine or the placebo, all returning eligible individuals were challenged with either rDEN2Δ30 at a dose of 10^3^ PFU (study 1) or rDEN3Δ30 at a dose of 10^4^ PFU (study 2). After both vaccine or placebo administration and virus challenge, the participants returned for follow-up every other day for 16 days and then on days 28, 56, 90, 180, and 360. Safety analysis was recorded up to 28 days after vaccination and challenge. All AEs were graded according to severity and relatedness. Solicited AEs included rash, retro-orbital pain, and headache. As part of the safety evaluation, blood was obtained for a complete blood count (CBC) with differential measurement and alanine aminotransferase (ALT) at regular intervals following vaccination and challenge.

For the determination of viremia, serum was evaluated every other day until day 16 after administration of either vaccine or placebo and subsequent virus challenge. Viremia was measured by amplification and direct titration in Vero cells, as previously described (27), with the following monoclonal antibodies used for serotype identification: 1F1 (DENV1), 3H5 (DENV2), 8A1 (DENV3), and 1H10 (DENV4). The lower limit of virus detection was 3 PFU/mL.

Neutralizing antibody responses to all 4 DENV strains in TV005 and rDEN2Δ30 were measured by 50% plaque reduction neutralization test (PRNT_50_), as previously described (27). PRNTs were performed before vaccine or placebo and challenge and on days 28, 56, 90, and 180 thereafter. The TV005 DENV2 vaccine component contains the prM and E genes from DENV2 NGC/1944 prototype strain (Asian II genotype), in which serum from the original 1944 patient was inoculated into a monkey to generate a serum pool (provided by Leon Rosen, NIH, Washington, DC) (35). This monkey serum was passaged 2 times in mosquito C6/36 cells and sequenced for generation of the cDNA to create the rDEN2/4Δ30 vaccine component. The DENV2 NGC target virus used in the PRNT assay is a passage 7 (p7) derivative of the original 1944 isolate (passaged in live monkey, C6/36, and Vero cells). The rDEN2Δ30 Tonga/74 challenge strain, an American genotype, is derived from 2 passages (p2) in C6/36 cells inoculated with patient serum from the 1974 outbreak in Tonga (provided by Duane J. Gubler, Duke-NUS Medical School, Singapore) followed by sequence analysis to generate the cDNA to create the rDEN2Δ30 challenge strain (GenBank accession AY744149). The DENV2 Tonga/74 target virus used in the PRNT is a p4 derivative of the original 1974 isolate (passaged in C6/36 and Vero cells) (20). DENV2 Tonga/74 has been shown to be more resistant to neutralization by serum from rDEN2Δ30-infected persons compared with other genotypes (29). Both DENV2 WT strains (NGC and Tonga/74) were used as target viruses for the PRNT of serum after vaccination and after rDEN2Δ30 challenge. The rDEN3Δ30/31 Sleman/78 vaccine component and rDEN3Δ30 Sleman/78 challenge strain are derived from p2 in C6/36 cells inoculated with patient serum from the 1978 outbreak in Indonesia (provided by Duane Gubler) (21) followed by sequence analysis to generate the cDNA to create the DENV3 Sleman/78 strains (GenBank accession AY656170). The rDEN3Δ30 Sleman/78 target virus used in the PRNT assay is a p5 derivative of the original 1978 isolate, which was engineered from the sequence derived from the C6/36 p2 isolate and subsequently passaged 3 times in C6/36 or Vero cells. Thus, the rDEN3Δ30 vaccine strain, challenge strain, and PRNT target strain share antigenic homology but differ in their attenuation phenotypes. Seropositivity was defined as a PRNT_50_ of 10 or greater; seroconversion was defined as a 4-fold or higher antibody titer compared with pre-vaccination (or pre-challenge) titers. Batching of the assays was done separately, with assays run for the post-vaccine period separately from the post-CHIM period. We defined infection following challenge as the presence of viremia or seroconversion by post-challenge day 90.

### Statistics.

A per-protocol analysis was performed. Fisher’s exact test was used to determine the protective efficacy of the vaccine after challenge with either rDEN2Δ30 or rDEN3Δ30, where viremia and rash in vaccine recipients were compared with those who received placebo. Fisher’s exact test was used to determine statistically significant solicited AEs in vaccinees compared with placebo recipients. For immunogenicity analysis, peak geometric mean titers were calculated, and the frequency of post-vaccine and post-challenge seropositivity was determined through day 90 following TV005 or placebo administration and challenge, and the frequency of challenge seroconversion through the study’s post-challenge day 90. A 2-tailed Student’s *t* test was used to compare the decline in clinical laboratory values. *P* values of less than 0.05 were considered statistically significant. Data in the figures are presented as individual responses along with the mean and 95% CIs, or the median and IQR where appropriate

### Study approval.

All research was approved by the IRBs of the University of Vermont Larner College of Medicine and Johns Hopkins Bloomberg School of Public Health. The TV005 vaccine and both virus challenge strains (DENV2, DENV3) were used under FDA Investigational New Drug (IND) applications (IND no. 15753). Informed consent was obtained from all participants, and the human experimentation guidelines of the United States Department of Health and Human Services were followed in the conduct of human research.

### Data availability.

Data supporting tables and figures are provided in the Supplemental Supporting Data Values file. The supporting computer code can be obtained by contacting the corresponding author.

## Author contributions

KKP, APD, SSW, SAD, and BDK conceptualized the study. APD, MC, DMD, SAD, and BDK conducted formal analysis. KKP, APD, MCW, BPS, SAD, and BDK conducted experiments. BDK wrote the first draft of the manuscript. All authors wrote, reviewed, and edited the manuscript. KKP and APD contributed equally to study design and conduct and share first authorship. The order of the co–first authors’ names was determined by agreement between the authors.

## Supplementary Material

ICMJE disclosure forms

Supporting data values

## Figures and Tables

**Table 9:**
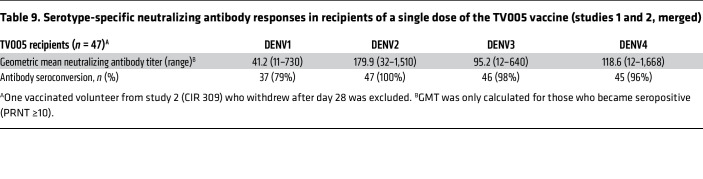
Serotype-specific neutralizing antibody responses in recipients of a single dose of the TV005 vaccine (studies 1 and 2, merged)

**Table 8:**
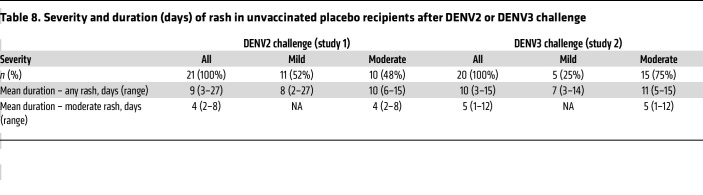
Severity and duration (days) of rash in unvaccinated placebo recipients after DENV2 or DENV3 challenge

**Table 7:**
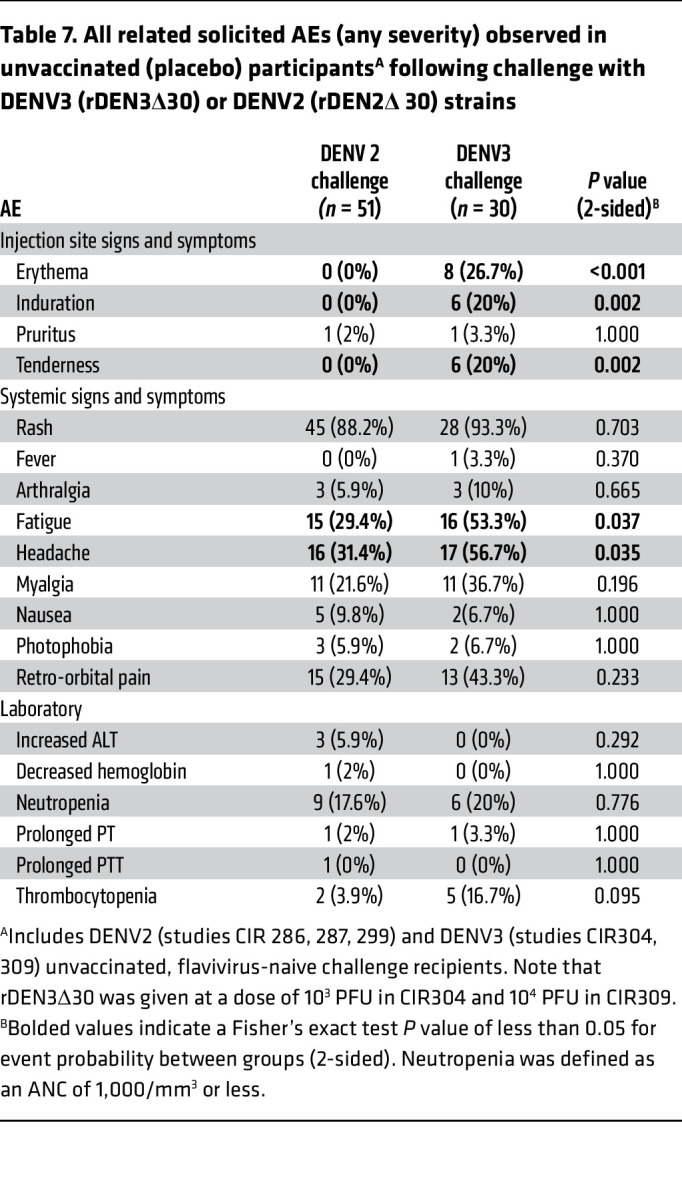
All related solicited AEs (any severity) observed in unvaccinated (placebo) participants^A^ following challenge with DENV3 (rDEN3Δ30) or DENV2 (rDEN2Δ 30) strains

**Table 6:**
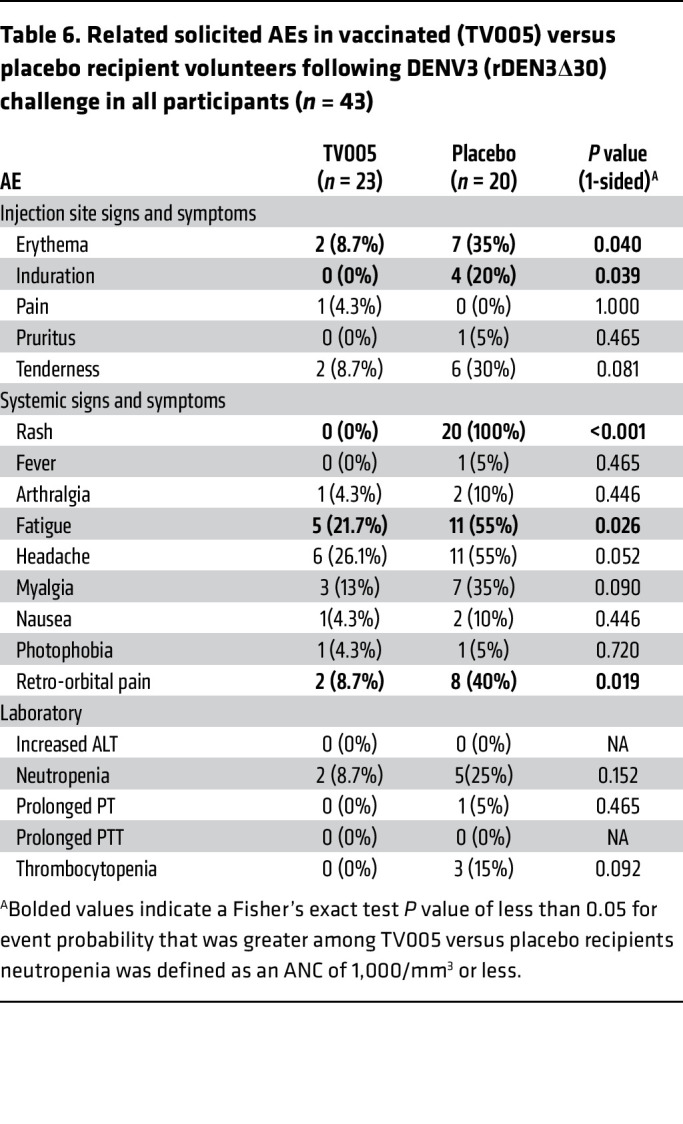
Related solicited AEs in vaccinated (TV005) versus placebo recipient volunteers following DENV3 (rDEN3Δ30) challenge in all participants (*n* = 43)

**Table 5:**
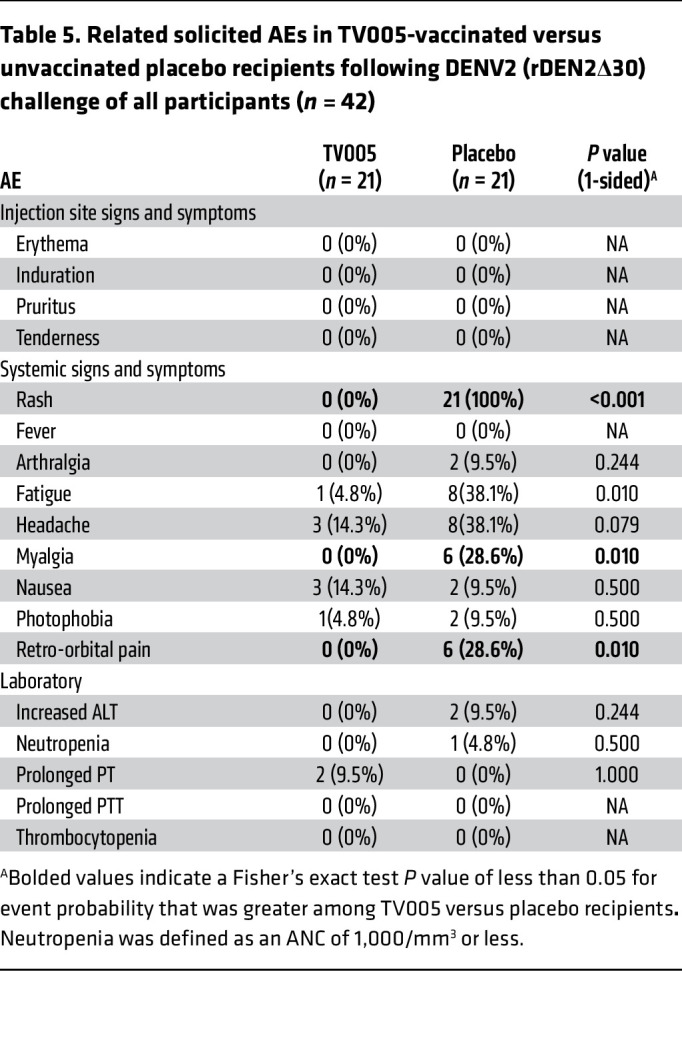
Related solicited AEs in TV005-vaccinated versus unvaccinated placebo recipients following DENV2 (rDEN2Δ30) challenge of all participants (*n* = 42)

**Table 4:**
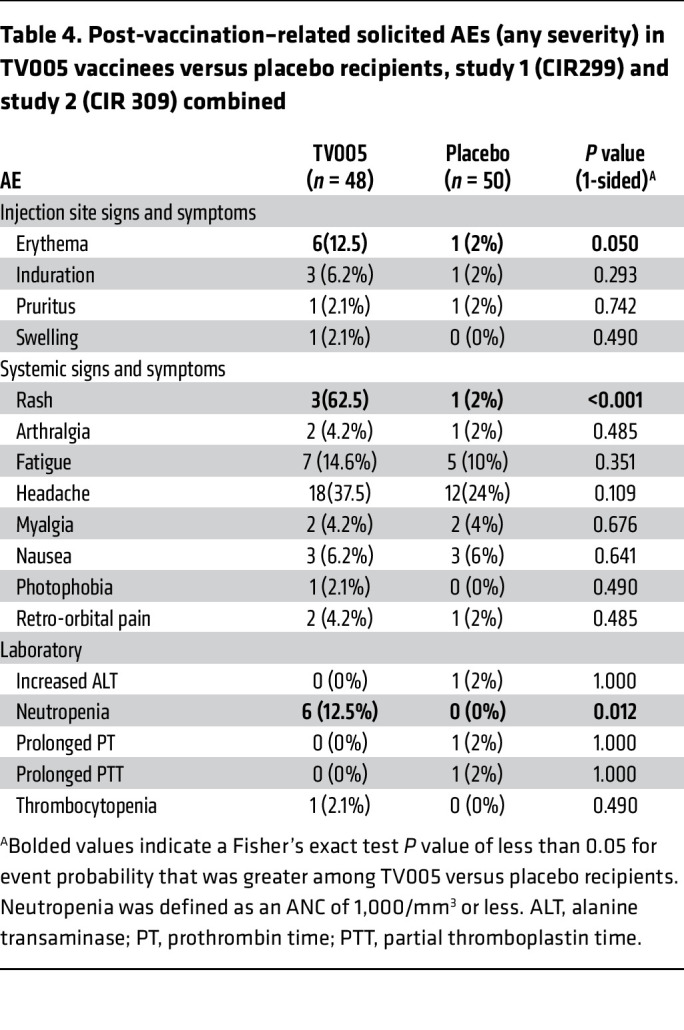
Post-vaccination–related solicited AEs (any severity) in TV005 vaccinees versus placebo recipients, study 1 (CIR299) and study 2 (CIR 309) combined

**Table 3:**
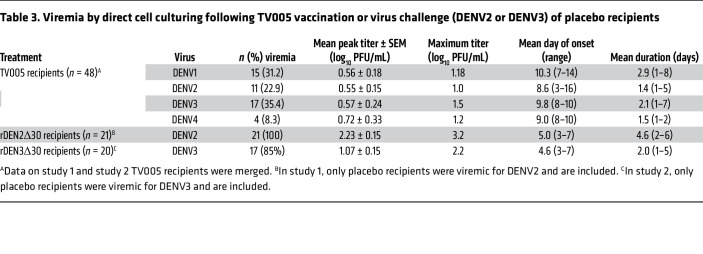
Viremia by direct cell culturing following TV005 vaccination or virus challenge (DENV2 or DENV3) of placebo recipients

**Table 2:**
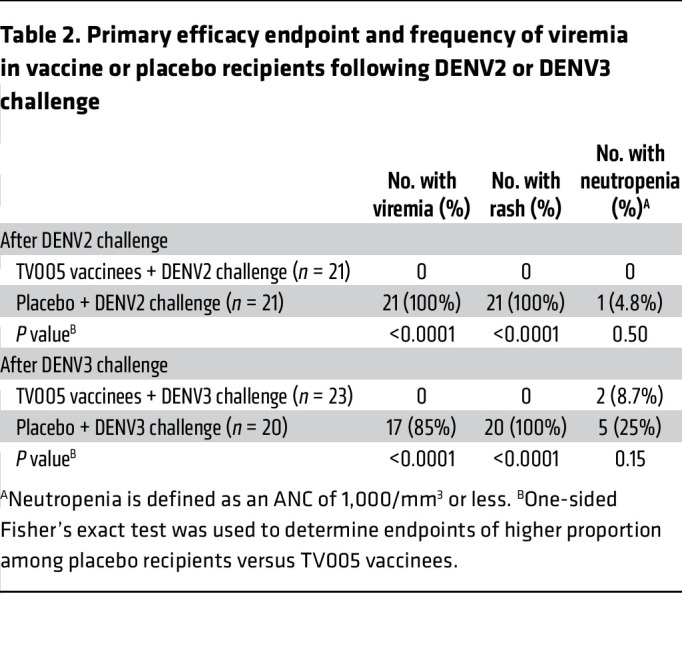
Primary efficacy endpoint and frequency of viremia in vaccine or placebo recipients following DENV2 or DENV3 challenge

**Table 1:**
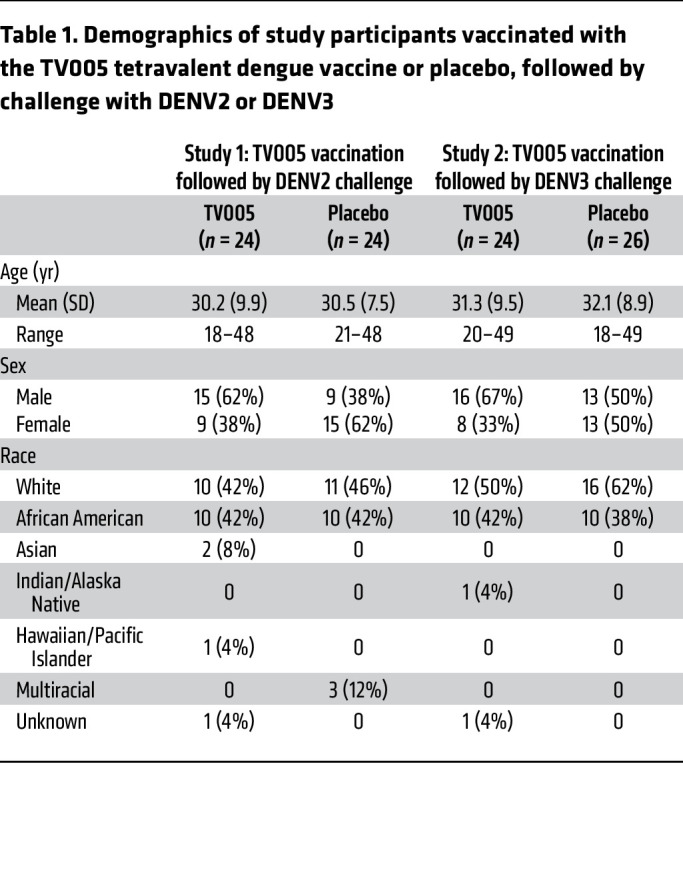
Demographics of study participants vaccinated with the TV005 tetravalent dengue vaccine or placebo, followed by challenge with DENV2 or DENV3

**Table 10:**
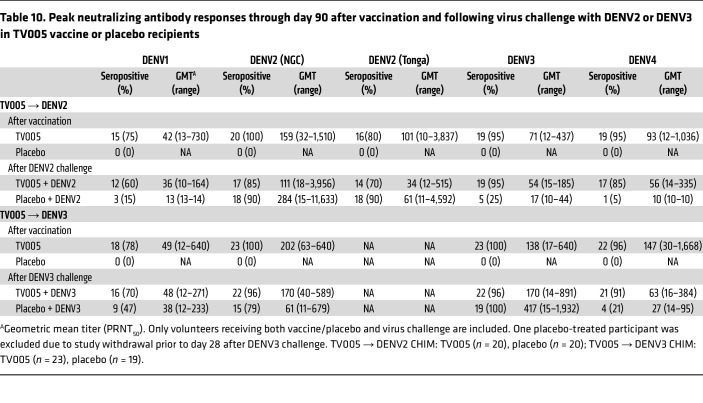
Peak neutralizing antibody responses through day 90 after vaccination and following virus challenge with DENV2 or DENV3 in TV005 vaccine or placebo recipients

## Version 1 11/16/2023

In-Press Preview

## Version 2 02/01/2024

Electronic publication
